# Lignin-Based Hydrogels for Sustainable Agriculture: Extraction, Design, and Applications

**DOI:** 10.1021/acsenvironau.5c00203

**Published:** 2026-03-02

**Authors:** Ankit Verma, Priyanka Devi, Sangeeta Bhogal, Rohit Jasrotia, Vijay Kumar Thakur

**Affiliations:** a Faculty of Science and Technology, ICFAI University, Baddi, Himachal Pradesh 174103, India; b School of Physics and Materials Science, 375605 Shoolini University, Solan, Himachal Pradesh 173229, India; c Biorefining and Advanced Materials Research Center, SRUC, Edinburgh EH9 3JG, United Kingdom

**Keywords:** lignin, extraction of lignin, extraction methods, structure of lignin, lignin-based hydrogels, synthesis of hydrogels, properties of hydrogels, agricultural applications of hydrogels

## Abstract

Synthetic polymers are widespread in modern life and pose growing environmental problems, especially in agriculture, where water management and soil health are crucial. Eco-friendly materials that balance performance and environmental safety are desperately needed as sustainable alternatives remain understudied. This study emphasizes the potential of lignin, a naturally occurring, abundant, and underutilized biopolymer, and its conversion into lignin-based hydrogels. Lignin hydrogels offer distinct benefits for agricultural applications due to their inherent antibacterial, biodegradable, and biocompatible properties. Their ability to swell improves soil water retention, promotes plant development in drought-prone areas, and permits regulated release of fertilizer. Lignin-based hydrogels can promote sustainable agricultural methods and lessen the dependency on synthetic polymers by customizing these characteristics. This study points to potential advances in green polymer technology by highlighting their capacity to bridge the gap between environmental stewardship and agricultural production.

## Graphical abstract

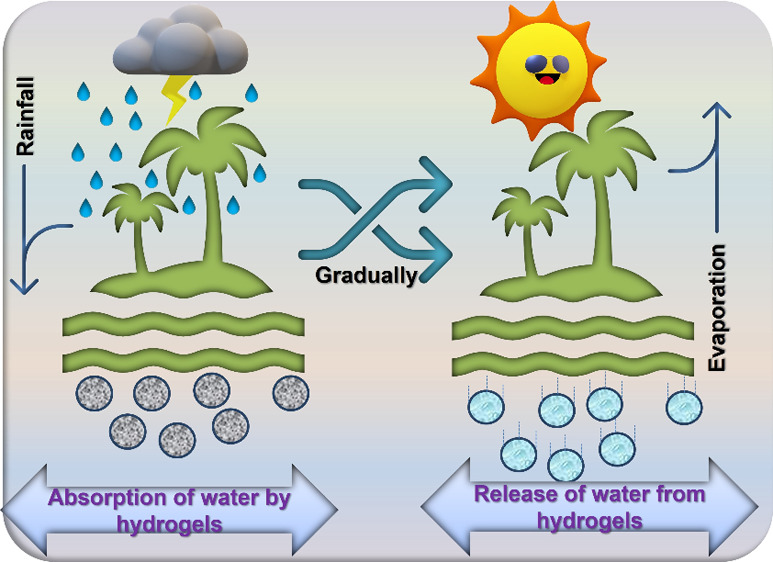

## 1 Introduction

Unfortunately, global health is under serious threat due to the exponential rise in hazardous pollutants entering ecosystems, driven by rapid industrialization, urbanization, and modernization.Such rapid progress has also caused environmental harm and adverse effects on human health. As these pollutants have entered our lives, they are affecting our health in unnoticeable ways. They have contaminated water bodies, soil and land, air, agricultural and food sources, and other essential requirements. In recent years, biodegradable resources have gained much interest. As a naturally occurring polymer, “Lignin” has drawn significant focus. Anselme Payen, the French chemist, used sodium hydroxide and nitric acid on wood in the 18th century.^1^ Two distinct products were obtained, one of which was cellulose, and the other was a carbon-rich material called “encrusting material”. This “encrusting material” was named as “Lignin” by Schulze, which means wood in Latin.^2^ Lignin is one of the three essential substances detected in a plant’s cell wall, which is essential for the structural and physical strength of the plant. Lignin is an amorphous natural polymer. It holds about 15–35% of the weight of lignocellulose biomass. Softwood contains about 30% of the total mass of lignin, whereas hardwood contains 20–25% of it. Next to cellulose, lignin ranks second as the most accessible natural aromatic material. Almost 30% of organic carbon in the biosphere comes from lignin. Lignin is produced and processed on a broad scale in the paper and pulp industries.^3^ As a substitute for fossil fuels, lignin is utilized to generate heat in the pulp industry. Lignin-based products exhibit good absorbance across the various materials. They can also be used in the synthesis of nanoparticles, photocatalyst, and supercapacitor electrodes. Lignin is a component of wood that is studied as a viable option due to its environmental friendliness, low cost, high abundance, and biodegradability. It can also be considered to be a competent adsorbent for wastewater treatment and a viable alternative to synthetic polymers. Because of the aforementioned attributes, lignin can be a viable option in the chemical industry .^4^ Such lignin traits help to improve environmental health and reduce reliance on synthetic polymers, thereby affecting human health. Due to the presence of numerous functional groups, it exhibits outstanding behavior in chemical reactions and modifications. These result in the formation of novel bioderived materials. The basic monomer units for lignin are sinapyl, coumaryl, and conferyl alcohols. Lignin is a naturally derived polymer with a complex structure that contains numerous polyphenols due to the polarity of several functional groups. It can also be utilized in chemical bonding as an advanced, sustainable biomaterial. The presence of diverse chemical components and complex 3D structures in lignin contributes to a variety of properties, including biodegradability, antibacterial and antifungal activity, stabilization effects, UV absorption, and reinforcing effects.^5^ It is not readily isolated or dissolved in organic solvents due to the presence of hydrophobic functional groups. Several methods have been employed to extract lignin from its sources. These methods include the kraft process, the soda process, the organosolv process, the sulfite method, and various other chemical methods. These techniques are discussed in Section 5 (Extraction Methods for Lignin).^6^ Hydrogels are three-dimensional complex networks, which are formed by hydrophilic polymers. Hydrogels are formulated by physical and chemical techniques.^7–9^ Polymer chains are created when multiple monomers interact with one another. These formed materials have the potential to absorb more water than their own weight while maintaining their original shape. Moreover, hydrogels from biopolymers exhibit high environmental compatibility.^10,11^ Nature-based polymers, such as polysaccharides and proteins, exhibit high biodegradability. This makes them a perfect option for forming biohydrogels. Because of lignin’s various attractive properties, it is a prominent component in the formation of biohydrogels.^7^ Lignin-based hydrogels are a promising alternative to synthetic hydrogels, owing to their biocompatibility. Physical and chemical methods are applied for the formation of lignin-based hydrogel.^9^ In the physical approach, lignin is blended or dispersed with the polymer.

Some chemical approaches to its synthesis include atom transfer radical polymerization, interpenetrating polymer networks, and reversible addition–fragmentation chain transfer polymerization. The variation in lignin quantity in its hydrogels affects the mechanical and tensile strength of lignin-based hydrogels, including storage modulus, rheological properties, and loss modulus.^5,12^

Lignin-based hydrogels have a wide range of applications. They can be used for water purification, oil drilling, agricultural purposes, the synthesis of biomedicines, and soil treatment to facilitate drug delivery, and as absorbent materials (Figure 1. However, their application to agricultural systems is of great interest. The lignin-based hydrogel exhibits various qualities, including biodegradability, eco-friendliness, antifungal activity, and nontoxicity. In a similar way, these hydrogels also have suitability with soil and do not degrade the soil’s quality. Owing to these traits, a lignin-based hydrogel can be utilized for numerous agricultural challenges. On account of the great swelling features of these hydrogels, they are useful to elevate the availability of water and enhance the flow of soil water in areas that are prone to drought.^14–16^ Hydrogels can additionally be employed as additives for the retention of water and conditioners for soils like sandy soil and silt loam soil.^17,18^ Hydrogels based on lignin embedded with additional polymers and nanoparticles can also be adopted for the preparation of an antioxidant and controlled-release fertilizer, which can enhance the activity for a longer duration.^19,20^ The lignin-based hydrogel can also be employed to exterminate the ions of heavy metals, such as Cd­(II) ions, from soil. They can also restrain the uptake of these toxic metals via the roots of plants.^20,21^ Hydrogels based on lignin show remarkable potential for additional applications in agriculture, thereby enabling a wide range of future research. Therefore, this review focuses on lignin-based hydrogels in agricultural implementations.

**Figure 1:**
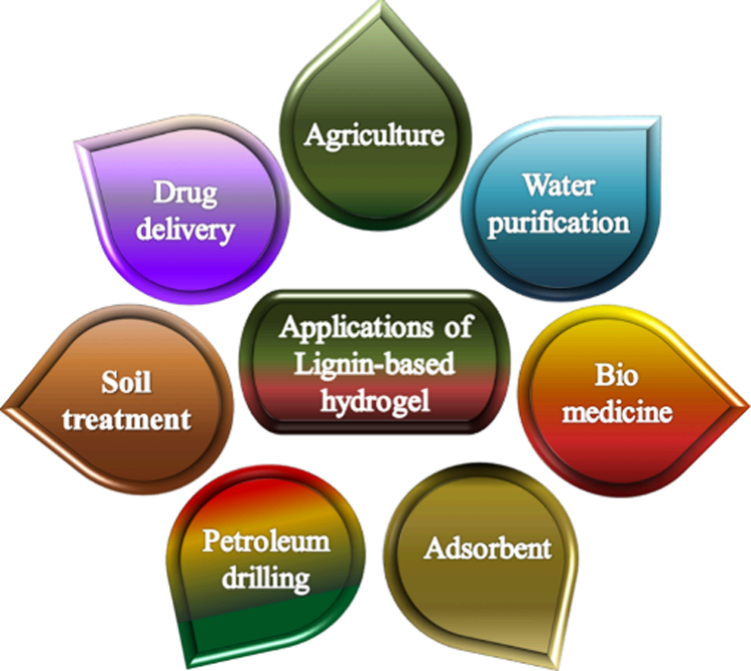
Overview of the different potential applications of lignin-based hydrogels demonstrating their importance in biomedicine, agriculture and environmental sustainability.^13^

## 2 Lignin

Lignin is present in the combined form of lignocellulose biomass. Biomass is composed of plant-based materials that are not used for food or feed. The natural polymers involved in lignocellulose biomass are hemicellulose, cellulose, and lignin. The biomass also contains some small amounts of ash.^22^ Lignin is a naturally derived polymer that has an amorphous structure. It is a biopolymer that ranks second to cellulose in terms of global abundance. Lignin in woody plant structures provides resistance to compression. It behaves as a waterproofing material for vascular elements, which plays a role in the conduction of minerals and water throughout the whole plant.^23^ The secondary plant’s cell walls are made up of lignin in combination with hemicellulose and cellulose. Lignin is water-insoluble and optically inert in nature.^6^ Many chemical and physical techniques are used to modify lignin to create various bio- and synthetic materials with better properties than the previous ones.^23^ The utilization of lignin as biofuel and other chemical products is shown in Figure 2.

**Figure 2:**
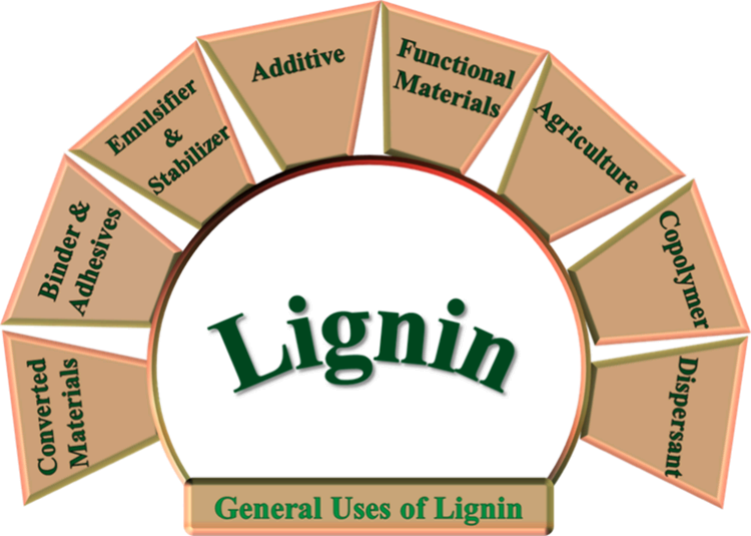
General uses of pure lignin in various fields, demonstrating its importance as a renewable and versatile biopolymer.^24^

Lignin contains carboxyl, carbonyl, methoxyl, and phenolic moieties in its large and branched 3D complex structure. This complex structure is capable of interacting with metal ions.^23^ Lignin is a biobased polymer with a complex structure. It is formed basically from three monolignols, which are 4-hydroxyphenylpropanoids. These monolignols are coniferyl, sinapyl, and *p*-coumaryl alcohols.^25,26^ These 4-hydroxy phenyl propanoids are different from each other because of their degree of methoxylation around the aromatic ring, which results in guaiacyl, syringyl, and *p*-hydroxyphenyl units in the context of lignin polymers.^27^

## 3 Source of Lignin

Lignin is next to cellulose, in terms of abundance. It is a biomacromolecule. The primary source of lignin is the plant’s cell wall, in the form of lignocellulose. “Lignum”, which means wood in Latin, was first named by Candolle.^6^ Lignin is one of the three major components of plant cell walls, which together form the plant cytoskeleton. The amount of lignin present in wood is about 19–35%, and in herbaceous plants it is about 14–24%.^28^ This biomacromolecule contains a large number of phenolic compounds, which have a molecular weight of 1 × 10^3^–2 × 10^4^ g/mol. Thus, it is regarded as the leading source of phenolic compounds. The linkage of cellulose and hemicellulose forms lignin–carbohydrate complexes. This linking is achieved through covalent and hydrogen bonds.^29,30^ This biomacromolecule is amorphous and is an aromatic biopolymer termed supermolecular self-assembled chaos.^6^ This supermolecule provides resistance to chemical attack, structural integrity against biological degradation, and rigidity in lignocellulosic biomass. Lignin plays a vital role in preventing external stresses from disrupting the cell walls of the structure.^31^ There is a well-known organic source on Earth: lignocellulose biomass. It is present in sufficient quantities to be used as a feedstock for the production of biofuel, bioethanol, biochemicals, and other valuable products.^6^ It primarily covers woody plants. The woody plants include softwoods (gymnosperms) and hardwoods (angiosperms). It also involves small amounts of sawdust, wood chips, sawmill waste, agricultural residues, bark, and similar materials. Lignocellulose-based materials and cell walls of plants consist of three main components. These are hemicellulose, cellulose, and lignin, with compositions of 15–35, 20–50, and 10–30%, respectively. Some are minor components, such as soluble sugars, minerals, proteins, and lipids, with respective compositions of 1–10, 5–10, 3–10, and 1–5%, respectively.^6^ Every year, the amount of lignin produced by plants is approximately 150 billion tons. Lignin is a biopolymer that has the highest carbon content. It is a carbon source with an estimated amount of approximately 95 billion tons.^32^ Various pulping methods are used by paper and pulp companies to separate tons of lignin from plant fibers. Lignin has several functional groups like phenolic groups, alcoholic groups, and carbonyl groups.^28^ As noted previously, lignin is produced in large quantities by paper and pulp companies as a byproduct. It is also used as a biofuel in place of fossil fuels. The amount of lignin consumed to produce heat is approximately 2% of 70 million tons.^33^ Lignin has a macromolecular structure composed of three main units connected by C–C and ether bonds. These units are syringyl (S), guaiacyl (G), and *p*-hydroxyphenyl (H).^34,35^ Lignin has difficulty tuning into microstructures. This is due to poor stability and weak interfacial interactions within the lignin. Because of its rigid structure, the lignin-based material is highly fragile. Thus, it has a poor functionality and mechanical properties. These limitations can be conquered by conventional fabrication and chemical modification methods.^36,37^ Lignin in normal wood primarily exists in the middle layer of the secondary cell walls of plants. The content and distribution of lignin can be influenced by external factors. Let us see for tension wood, the content of lignin in the secondary cell walls is less than that of normal wood, and the curving or sloping of branches and trunk affects the tensile strength of the xylem.^35^ However, the lignin content in the middle layer of secondary cell walls is much higher for compression woods than that for normal ones. This is due to high lignification in natural woods.^35^ Gymnosperm lignin is generally built of G units, and there is a shortage of S units. Thus, it has more branches than angiosperm lignin, which is rich in S and G units. Gramineous lignin has all of the S, G, and H structural units. It also contains ferulates and *p*-coumarates in abundance in the cell walls. These groups are linked to the phenylpropane structure via chemical linkage.^35^ The structure of lignin varies with its relative abundance in plants.^38^ Examples are wheat stems and leaves: Here, wheat straw stems have a lignin content of about 23.8%, whereas wheat straw leaves have a lignin content of about 16.6%.^39,40^ The lignin present in the leaf is more than that of the stem.^35^

## 4 Reported Waste Materials for Lignin Extraction

Various sources of lignin for extraction have been identified in studies, including woody plants and agricultural residues. Some documented raw materials used for lignin isolation are listed in Figure 3. These sources are extensively documented in the literature, highlighting the diversity of materials available for lignin production. The kind of plant material utilized can greatly impact the characteristics and extraction techniques of lignin. Some sources of lignin are listed below.

**Figure 3:**
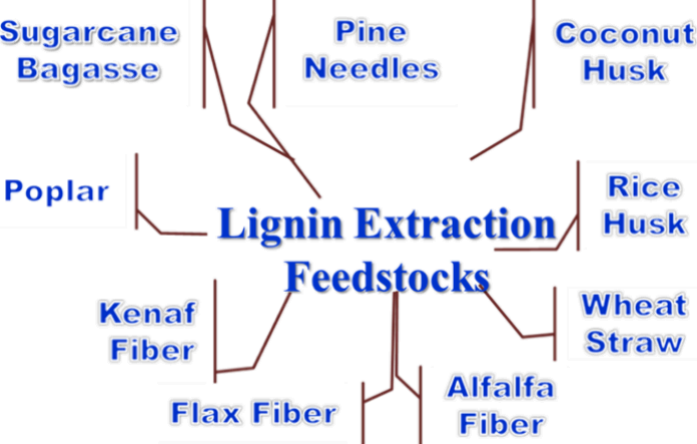
Different raw materials identified as key sources for lignin isolation and supporting sustainable biorefinery approaches.^45–47^

### 4.1 Extraction from Coconut Husk

Lignin can be extracted from coconut husk using various methods, such as soda pulping, organosolv pulping, alkaline pulping, and kraft pulping. Lignin produced by these methods has a diverse range of applications. According to Latif et al. and Lebedeva et al., this lignin can be employed as a nature-based diesel fuel and as a lubricant base oil, showcasing its potential in renewable energy and industrial lubrication sectors.^41,42^ Furthermore, Kumar and Kumar have demonstrated that the extracted lignin can effectively remove Cr­(V) ions from water, highlighting its application in water purification.^43^ Additionally, kraft pulping generates kraft lignin, which is effective in removing heavy metal ions from industrial effluents, as reported by Baraskar et al.^44^ This indicates its significant role in environmental remediation, particularly in treating wastewater from industrial sources. In essence, isolating lignin from coconut husk provides a sustainable lignin supply and enables a range of applications in industrial lubricants, biofuel production, water purification, and environmental cleanup.

### 4.2 Extraction from Rice Husk

Rice husk is another significant resource for lignin extraction. Numerous techniques are used to extract lignin from rice husk, including alkaline extraction, soda-ethanol treatment, organosolv pulping, alkaline hydrogen peroxide treatment, and alkaline peroxide treatment.^48^ Each approach assists in the separation of lignin from the cellulose and hemicellulose components of rice husk. This separated lignin can be applied to various applications. This extracted lignin has a noteworthy use in the elimination of nickel metal ion from contaminated water.^49^ By implementing lignin derived from rice husk for the removal of nickel ions, this study highlights its potential as a cost-efficient and renewable solution for the treatment of industrial effluents. This addresses the concern regarding the disposal of rice husk and provides a renewable resource for mitigating heavy metal contamination in water. In summary, the isolation of lignin from rice has significant applications in environmental restoration, particularly in the extraction of heavy metal ions from polluted water. This underscores the dual advantages of using agricultural waste, i.e., rice husk, as a source for lignin production and as an effective solution for water pollution.^50^

### 4.3 Extraction from Wheat Straw

Another significant raw material for lignin extraction is wheat straw. Several methods are used to obtain different types of lignin from wheat straw, including organosolv, kraft, and soda pulping, yielding organosolv, kraft, and soda lignin, respectively. Each one of these types of lignin has exceptional traits and potential utilization. Lignin isolated from wheat straw has notable applications as a lithium-ion binder. According to studies by Ramezani and Sain and Dominguez-Robles et al., lignin can elevate the stability and functionality of lithium batteries, which results in a promising material for energy storage technology.^51,52^ Moreover, lignin derived from wheat straw via alkaline extraction displayed antioxidant properties. Research by Ma et al. showed that this type of lignin can be employed for various implementations where antioxidant activity is advantageous, like in cosmetics, food preservation, and pharmaceuticals.^53^ The isolation and utilization of wheat straw-derived lignin provide several benefits. It offers a sustainable approach to utilizing agricultural waste, thereby reducing reliance on nonrenewable resources. The antioxidant and binder properties of lignin derived from wheat straw in lithium batteries highlight its potential across various industries.

### 4.4 Extraction from Pine Needles

The kraft pulping process can be employed to extract lignin from pine needles. This method can effectively separate lignin from cellulose and hemicellulose constituents of pine needles and yield kraft lignin, which has several industrial applications. The noticeable use of lignin derived from pine needles is as a biofuel. The high-energy constituents of lignin make it a great choice for the generation of renewable energy, which supports sustainable energy approaches and decreases our dependency on fossil fuels.^54^ Additionally, lignin derived from pine needles has demonstrated an effectiveness in removing dyes from wastewater. The studies of Kumari et al. showed that this lignin can adsorb and remove dyes like malachite green and methyl orange.^55^ This property of lignin is particularly beneficial for treating industrial effluents, where dye contamination is a major environmental concern. The application of lignin derived from pine needles for the removal of dyes demonstrates its potential for environmental remediation. Lignin acts as an adsorbent to help reduce pollution, purify water, and mitigate the harmful effects of synthetic dyes in aquatic ecosystems.

## 5 Extraction Methods for Lignin

Lignin is extracted from plant fibers, as discussed in Section 6. However, the isolation of lignin has its own challenges.^56^ The complex cell wall structure and the interactions among lignin components make it difficult to isolate lignin from plants.^56^ To break these interactions, some chemical methods must be taken into use, as lignin is present in lignocellulose biomass.^56^ Bjökman was the first to extract lignin with the help of a dioxane–water mixture in 1956.^6^ After that, many processes emerged to isolate and separate lignin from the lignocellulose biomass. The lignin produced has high purity and a less complex structure.^6^ Two methods were identified for extracting lignin from plant fibers.^56^

First, the enzymes are used to separate lignin from carbohydrate by dissolving carbohydrate, while lignin remains unaffected and as it is an insoluble product.^56^ This includes a typical approach in the biorefinery concept, in which feedstock is pretreated to break the bonds between carbohydrates and lignin. Several pretreatment methods are available to extract high-quality lignin from various sources. Some of these methods are alkali treatments, acid treatments, mechanical grinding, ball milling, and enzymatic hydrolysis.^56^ These methods are mainly categorized as chemical, physicochemical, and enzymatic methods. In this process, the physical structure of biomass is dismantled, and the size is diminished.^56^

### 5.1 Chemical Methods

This method involves the pretreatment of biomass with various chemicals, such as alkalis, acids, organic solvents, reductive reagents, oxidants, and ionic liquids.^57^ The most widely used chemical pretreatment method is an acidic treatment. During acid treatment, the process of breaking the bonds between carbohydrates and lignin also partially breaches other bonds such as lignin–lignin ether bonds. Initially, with the breaking of β–O–4 bonds, lignin gets depolymerized, and simultaneously a new C–C bond is formed because of an attack on aromatic rings by an electron-deficient carbon ion. A new lignin matrix with a condensed phase is produced because the depolymerization of lignin is reversed as a result of the occurrence of a new C–C bond. However, sufficient lignin–carbohydrate complexes can be generated by decreasing the solvent’s acidity. This is done so the lignin structure can be avoided from being degraded.^56^

In addition to acidic treatment, ionosolving methods are available. In this chemical method, ionic liquids are used. The ionic liquids are salts that, in particular, under certain conditions, exist in a liquid state. It contains organic or inorganic ions. It has favorable physicochemical properties, including low volatility, a depressed melting temperature, enhanced thermal stability, and reduced flammability. By mixing ionic liquids with organic solvents and water, their dissolution and viscosity properties can be modified. Even the selectivity of ionic liquids toward the extraction of lignin from biomass can also be modified.^58^ The high cost of their production and toxicity problems make the use of ionic liquids complex in biorefinery.^59^

### 5.2 Physico-chemical Methods

This method involves mechanical grinding, hot water, steam explosion, ammonia fiber expansion, and pyrolysis treatments.^32,57,60^ In mechanical grinding pretreatment, the structure of lignin can be altered by a growing amount of carbonyl and phenolic-hydroxyl groups. Breaking the β–O–4 bond can also change its chemical structure.^56^ The most promising pretreatment is a steam explosion. It is a thermochemical process that converts biomass into fractions. This pretreatment is environmentally friendly and works with low energy consumption. The investment cost and use of hazardous chemicals are also less in the stem explosion pretreatment.^61^

### 5.3 Enzymatic Methods

The enzymatic pretreatment method has merits such as eco-friendliness, cost-effectiveness, and a high likelihood of degrading plant cell materials. The polysaccharides of lignocellulose biomass are degraded by using hemicellulose and cellulose in enzymatic hydrolysis.^40^ The isolated lignin does not react with enzymes and is known as hydrolysis lignin. This obtained lignin can be employed for various goals, for instance, resins, sorbents, and polymeric chemicals.^6^

In other words, black liquor, which contains lignin, is obtained in the process. The lignin produced by the second method is classified into two main categories (Figure 4).

**Figure 4:**
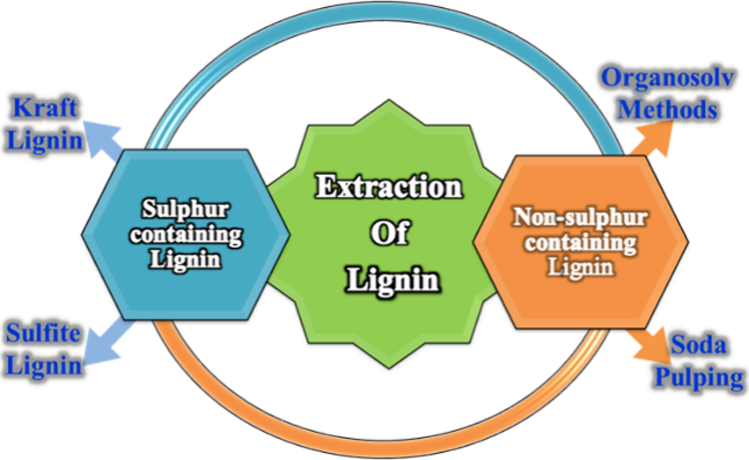
Schematic overview of major lignin extraction routes, highlighting sulfur-containing and nonsulfur-containing pulping processes.^62^

- Sulfur-containing lignin
- Non-sulfur-containing lignin

The commercial availability of sulfur-containing lignin is more than that of nonsulfur lignin.^56^ The lignins produced are different from each other based on their structures, reactivity, and efficiency, even if their source is the same. This happens because of various methods used for extraction.^6^

### 5.4 Sulfur-Containing Lignin

The lignins involved in sulfur-incorporating lignin are kraft lignin and sulfite lignin. The primary sources for these lignins are papers and pulp companies.^6^

#### 5.4.1 Kraft Lignin

Among the major methods for lignin extraction in the pulp and paper industry is kraft pulping. In this technique, lignin is extracted from the lignocellulose biomass. The kraft process was initially developed in Poland in 1879. Currently, about 55–90 million tons of lignin is formed from 130 million tons of kraft pulp per year. This process is performed in aqueous solution using NaOH and Na_2_S at 170 °C for approximately 2 h. In the kraft process, the cleavage of ether linkages increases the number of phenolic–OH groups. The presence of Na_2_S causes depolymerization of lignin and decreases the degradation of carbohydrates. The alkaline medium-ionized phenolic groups present in lignin, which induce the formation of dark pulp, and lignin gets dissolved in water.^56^ The soluble fraction produced by depolymerization is called kraft lignin. This lignin can be isolated at 5–7.5 pH from black liquor.^6^ Lignin is produced in a high yield of about 90–95 wt %. Lignin is broken down by kraft pulping, and the lignin molecular weight is between 1500 and 25,000 g per mole.^58^

#### 5.4.2 Sulfite Lignin (Lignosulfonates)

Among the other ways for the extraction of lignin from lignocellulose biomass, the sulfite pulping method is the oldest way. Approximately 1 million tons of commercial lignin are produced annually. This process takes place in the presence of sulfite salts like magnesium sulfite, calcium sulfite, sodium sulfite, and ammonium sulfite at a temperature of 140–180 °C for 1–5 h. The process occurs at pH = 2–12. The sulfite process is feasible in all three media, i.e., acidic, alkaline, and neutral. In the acidic process, magnesium or calcium sulfite is used; in the alkaline process, ammonium or sodium sulfite is used. The lignosulfonates are highly soluble in water and can be separated from carbohydrates, which form a dark pulp. The sulfite lignin can be recovered in three steps. In the first step, a complex is formed in which a long chain of alkylamines is attached to it. In the second step, the organic solvent is used to extract the complex, and in the last step, an alkaline is used to regenerate the sulfite lignin.^58^ The obtained sulfite lignin has a higher average molecular weight than that of kraft lignin.^63^ The lignin extracted by the sulfite pulping method is mainly a mixture of inorganic materials, ashes, and carbohydrates.

### 5.5 Non-sulfur-Containing Lignin

The nonsulfur-incorporating lignin is organosolv lignin and soda lignin. These extracted lignins can be employed as phenol components or low molecular mass aromatic components due to their features.^6^

#### 5.5.1 Organosolv Methods

The organosolv method is an environmentally friendly technique for lignin recovery compared with the kraft or sulfite processes. This process uses organic solvents, such as ethanol, methanol, ethers, acetone, polyols, or their mixtures, to solubilize lignin and hemicelluloses. The sulfurous chemicals are not solubilized as they produce huge amounts of organosulfur products.^58,64,6^ Organic solvents used in this process generally have low boiling points; thus, they are easy to remove and recycle. This process can be used with or without acidic, basic, and mineral catalysts at temperatures of 100–250 °C for 30–90 min.^58,65,66^ During lignin extraction, the organosolv process is effective at preserving the native lignin structure. This method is efficient at preferentially separating lignin–carbohydrate bonds, and lignocellulose biomass can also be separated into lignin, hemicellulose sugars, and cellulosic fibers.^45,47^ This technique produced the purest lignin with the lowest carbohydrate content. The organosolv method has limited global reach because the recovery of organic solvents is expensive and equipment is corrosive.

A technique, which was designed by Watkins et al., for the extraction of lignin by using the organosolv process, is discussed by Zourif et al., with some modifications.^67^ In this study, *Reseda luteola* L. waste was used for the isolation of lignin after extracting its color (Figure 5a). The methodology involved mixing 10 g of *Reseda* waste powder with an organic acid mixture (30:70 v/v) of acetic and formic acids in a conical flask, yielding a mass concentration of 75–99% and integrating the mixture into biomass. The ratio for liquid/fiber was between 10:1 and 4:1. The prepared mixture was brought to a boil in a reflux system for 2 to 4 h. The components were then cooled to room temperature. Separation of fibers was done using a Buchner funnel. To get the lignin-rich filtrate, the fibers were first rinsed with 80% formic acid and afterward with warm distilled water. Then, the fibers were delignified using a mixture of peroxyformic acid/peroxyacetic acid solutions. To make this mixture, 8 mL of 35% hydrogen peroxide was added to the organic acid, which had a mass concentration of 75 to 99%, a blend of acetic acid and formic acid, and then heated at a temperature of 80 °C for 2 to 4 h. To separate the delignified fibers from the lignin-rich filtrate, the mixture was then filtered and rinsed with hot water. The precipitates of lignin were obtained by introducing distilled water five times greater than the quantity of concentrated liquor and filtered using a Buchner funnel, followed by washing with distilled water. The obtained lignin was then oven-dried at 50 °C to achieve a stable mass. The extraction of lignin is depicted in Figure 5b.

**Figure 5:**
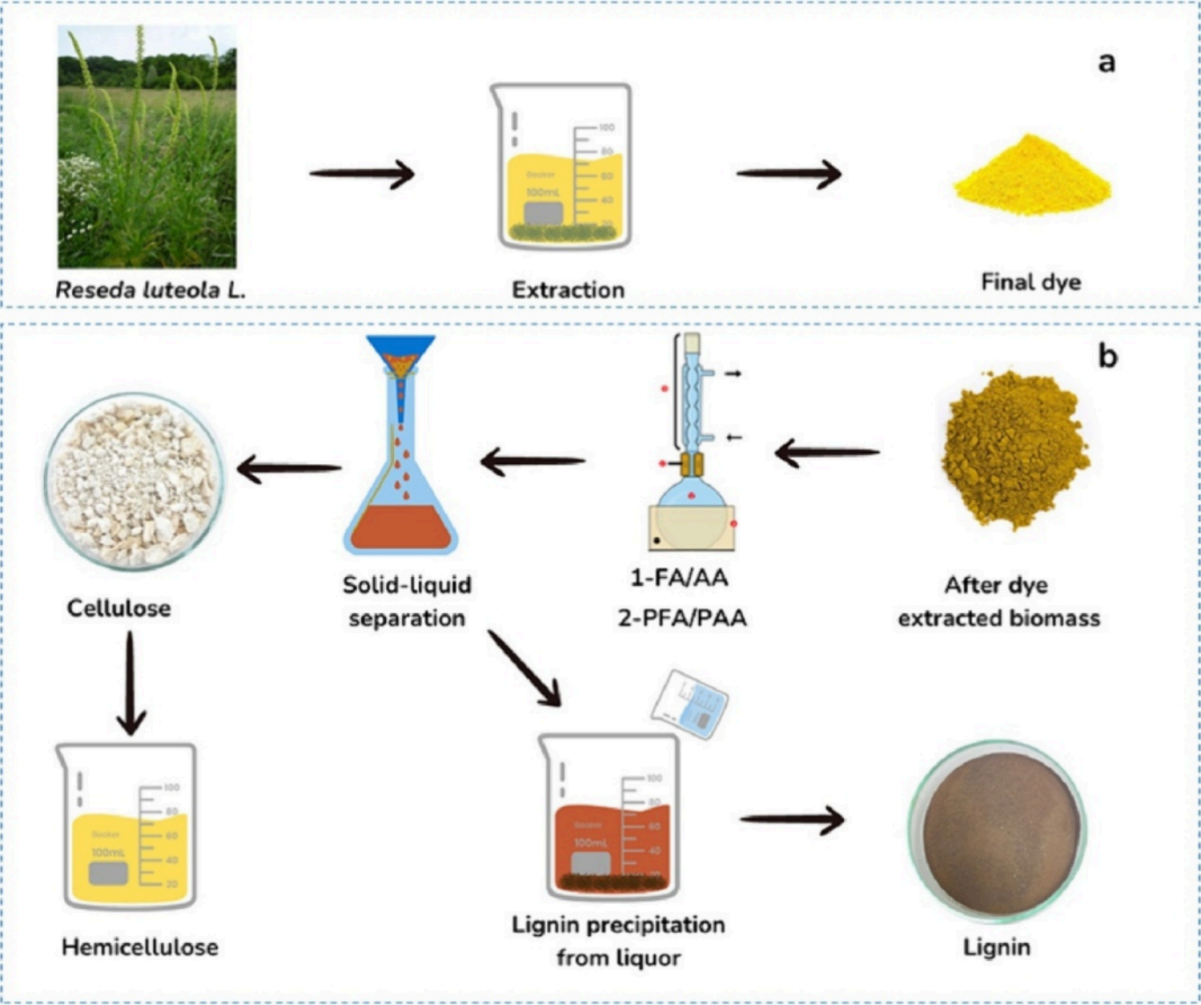
(a) Extraction of color from *Reseda luteola* L. (b) Extraction of lignin from *R. luteola* L waste. Reproduced with permission from ref 67. Copyright 2024 Elsevier.

#### 5.5.2 Soda Pulping

The soda pulping method is among the main processes that produce pulp from nonwoody biomass with low lignin content, such as herbaceous plants like bagasse, wheat straw, hemp, grass, sugar cane bagasse, sisal, flax, and kenaf. In this process, NaOH (sodium hydroxide) is used to solubilize lignocellulosic biomass in the thermal range from 140 to 170 °C at high pressure.^36^ The alkaline soda pulping process is somewhat similar to the kraft process. However, it proceeds without Na_2_S because the depolymerization of lignin is inefficient. The depolymerization of lignin starts with breaking β–O–4 bonds in phenolic groups and then breaking in nonphenolic groups. As a result of these reactions, free phenolic groups are generated. These groups are converted into ions, and the lignin starts to dissolve in the alkaline solvent.^56^ The soda lignin recovered after much effort through filtration or centrifugation processes has a significant proportion of carboxylic acid.^6^ However, the lignin obtained is free from sulfur. Thus, it can be used for several implementations, like the synthesis of bioplastic and composites.^68^ The average molecular weight of the obtained soda lignin falls between 1000 and 3000 g/mol.^58^

The main characteristics, benefits, and drawbacks of the various pulping techniques covered are highlighted in Table 1, the comparative table below. It provides a comprehensive side-by-side comparison to assess its efficacy, sustainability, and suitability for lignin extraction.

**Table 1:** Comparison of Pulping Techniques Utilized for the Extraction of Lignin

extraction process	kraft process	sulfite process	organosolv process	soda process
**lignin**	kraft lignin	sulfite lignin	organosolv lignin	soda lignin
**raw material**	hardwood and softwood	mainly softwood	lignocellulosic biomass	nonwood biomass
**Chemicals used**	sodium hydroxide and sodium sulfide	sulfite salts	organic solvents and acid catalyst	sodium hydroxide
**mechanism**	cleavage of ether bonds	sulfonation of lignin	delignification by NaOH	lignin becomes soluble in an organic solvent
**Medium of solvent**	alkaline	acidic, neutral and alkaline	acidic	alkaline
**working condition**	170 °C	140–180 °C	100–250 °C	140–170 °C
**environmental concern**	sulfur-containing lignin	sulfur-containing lignin	sulfur-free lignin	sulfur-free lignin

## 6 Structure of Lignin

The lignin in lignocellulosic biomass is made of three elements, i.e., C, H, and O. The free radical polymerization of the phenyl propanol unit gives rise to lignin. The polymer unit of lignin, which mainly exists in the secondary plant’s cell walls, imparts its rigidity.^35^ The free-radical coupling of the phenylpropanol monomer introduces heterogeneity into the lignin. The lignin has three phenylpropane monomers in its backbone. These are *p*-coumaryl, sinapyl, and coniferyl alcohols. These monomers are building blocks for secondary cell walls in plants. Several C–C and C–O bonds are present amid these monomers, such as 5–5, 4–O–5, β–β, and β–O–4. Among these, linking β–O–4 is most common.^56,69^ The configuration type of the lignin molecule depends on the portion of these three monomers. The configuration type illustrates the magnitude of branching involved and the reactivity of lignin.^70^ These phenolic monomers have other substructures. They are named *p*-hydroxyphenyl (H), syringyl (S), and guaiacyl (G) (Figure 6). There are several functional groups, such as carboxyl (COOH), carbonyl (C–O), hydroxyl (OH), and methoxyl (OCH_3_). The mentioned functional groups are involved in chemical alteration of lignin to enhance its uses.^71^

**Figure 6:**
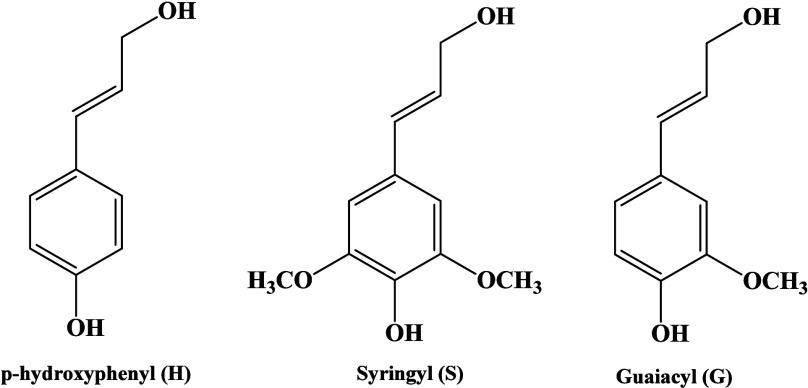
Chemical structure of guaiacyl (G), syringyl (S), and *p*-hydroxyphenyl (H).^67^

There is no particular structure for lignin. This is because the lignin structure varies with different extraction techniques. It also depends upon the biomass used for extraction and the circumstances for its growth.^72^

For the very first time, Klason clarified the chemistry of lignin in 1897. He hypothesized that lignin was formed by coniferyl alcohol units, which was later confirmed by characterization methods.^38^ In 1875, Tiemann and Mendelsohn isolated coniferin, which is a glycoside of coniferyl alcohol, from the flowing substance of the cambial layer that was still growing. In 1908, it was suggested that lignin had a complex molecular structure.^38^ Alder, Freudenberg, and Brunow et al., in respective years 1977, 1965, and 1998, formed a structure for softwood lignin, whereas Nimz in 1974 gave a structure for hardwood.^73^ The chemical composition of lignin is demonstrated in Figure 7.

**Figure 7:**
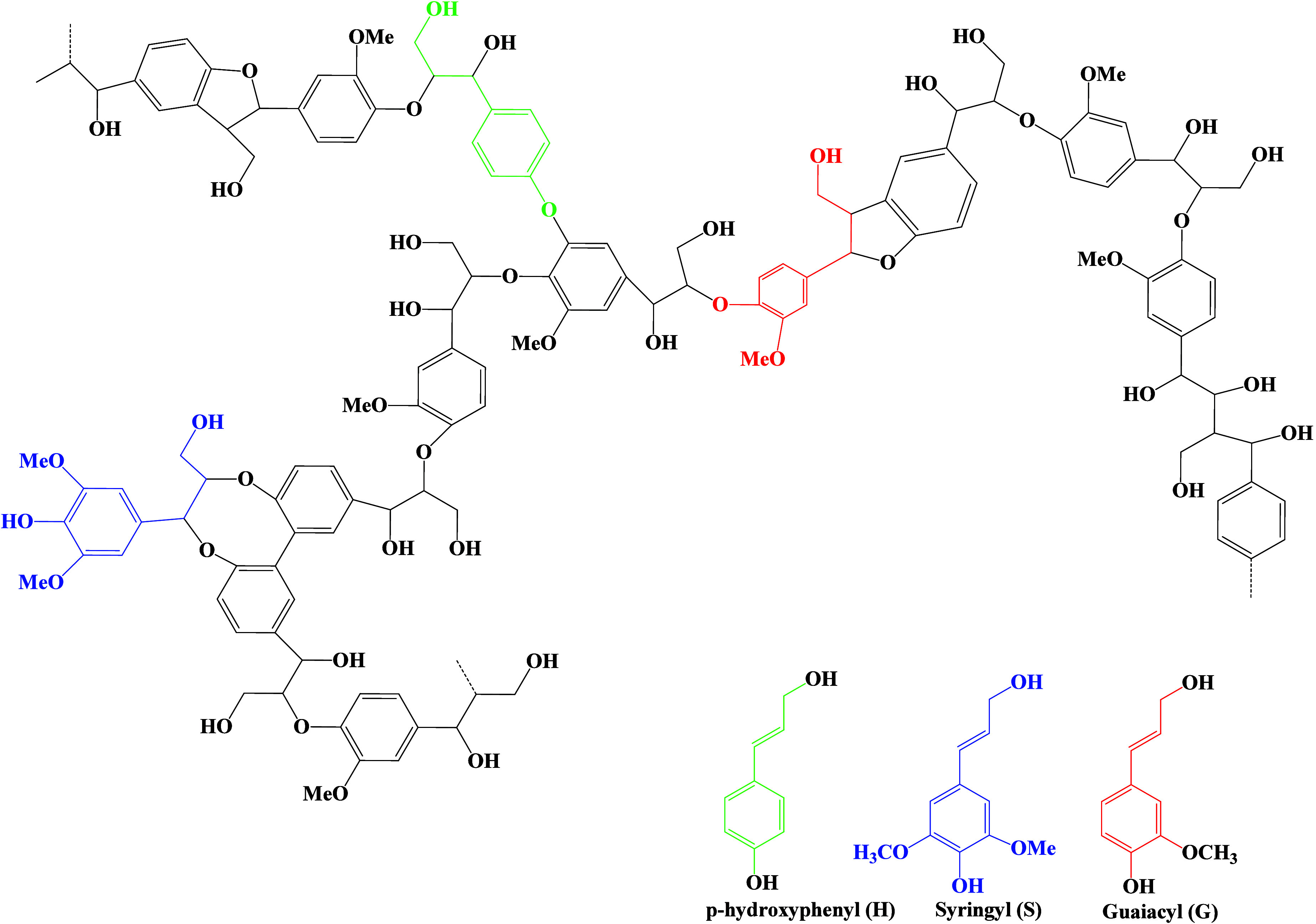
Overview of lignin chemical composition highlighting the functional groups that enable its diverse applications.^74^

Each phenylpropanol monomer has an aromatic group and a chain of three carbons. These carbons are named as α, β, and γ. Lignin has a number system for its groups. The *p*-hydroxyphenyl unit lacks a methoxyl group, and the syringyl unit contains two aryl methoxyl groups; however, the guaiacyl unit contains only one aryl methoxyl group. The presence of these groups at positions 3 and 5 on the aromatic rings is because of the degree of substitution.^6^ The lignin structure, obtained from angiosperm monocot, angiosperm dicot, and gymnosperm wood, was studied by Ralph et al. Softwood lignin of gymnosperm wood contains coniferyl alcohol, which produces guaiacyl (G) subunits. A total of 20 monomeric units of the G subunit are present in gymnosperm wood. It also contains one unit of each spirodienone, resinol, 4–O–5 biphenyl ether, 5–5/4–O−β dibenzodioxocin, 12 β–O–4 linkage in β-ether, and two end groups of cinnamyl alcohol. The angiosperm dicot hardwood lignin has a total of 20 monomeric units. Thirteen of them are syringyl (S) subunits, and seven of them are guaiacyl (G) subunits. It also contains one unit of β–5 phenylcoumaran, ether, 4–O–5 biphenyl resinol, 16 β–O–4 linkages in β-ether units, and cinnamyl alcohol group. Dibenzodioxocin and spirodienone units are not present in it. The angiosperm monocot lignin also contains 20 monomeric units. Six of them are guaiacyl (G) subunits, and 13 are syringyl (S) subunits. It also consists of one unit of 4–O–5 biphenyl ether, tricin unit, and tetrahydrofuran. Two units of β–5 phenylcoumarans, 15 units of β–O–4 β-ether, and 2 γ-acetates are also present. However, spirodienones, dibenzodioxocins, and cinnamyl alcohols are unavailable. Angiosperm monocot lignin includes more acetates than angiosperm dicot lignin does. In gymnosperms, lignin acetates are absent.^75^

The lignin compositions are different for different plant tissues. According to the characterization of the structure of lignin, it is shown that the syringyl (S) unit and aryl ether bonds are in greater amounts in stem lignin than in leaf lignin. The stereochemistry of lignin has revealed that its side chain adopts different conformations. The simple organic acids can be produced by converting part of the side chain of lignin by ozonation treatment, while double bonds and aromatic groups are destroyed. However, nothing has changed in the actual stereo structure of the lignin side chain. Information about the stereo configuration or structure of the lignin side chain is acquired by ozonation treatment methods.^76,35^ Various kinds of treatments, like chemical, biological, and physical, can change the lignin structure.^35^

## 7 Lignin-Based Hydrogel

Hydrogel is a complex 3D network of synthetic and natural polymer chains that tend to absorb and retain liquids without undergoing solubilization. The polar hydrophilic groups of hydrogels show hydrating properties, which give them the capability to swell. The majority of the widely available hydrogels are prepared from synthetic polymers. These synthetic polymers are petroleum-based and are not compatible with the environment, with a low rate of biodegradation. The cost of preparing these petroleum-based polymers is relatively high, and the rate of water absorption from concentrated electrolyte solutions is low.^77^ Because synthetic polymers have several drawbacks, the use of natural polymers in the preparation of hydrogels has gained increasing interest. Because biopolymer-based hydrogels are compatible with the environment, they are biodegradable. As a result, they are used for biomedical purposes, as well as in foods, cosmetics, and pharmaceuticals.^78^ Moreover, some of these biopolymer-based hydrogels may exhibit limitations in their mechanical properties. Natural polymers that can be used to produce hydrogels include cellulose, hemicelluloses, chitosan, collagen, lignin, proteins, gelatin, starch, sodium alginate, hyaluronate, and their derivatives.^79,80^ Lignin can be used to synthesize natural-based hydrogel.^69^ Lignin contains phenylpropane units and other functional groups such as carbonyl, hydroxyl, phenolic, and aliphatic groups. The hydrophilic groups present in the lignin structure contribute to its suitability as a material for hydrogel preparation. The functional groups are helpful for forming bonds with water and for chemical reactions and modifications.^81,82^ As a biopolymer, lignin exhibits properties such as biocompatibility, biodegradability, nontoxicity, antimicrobial activity, and antioxidant activity. Therefore, it is a notable source for the formation of water-absorbent hydrogels that can be petroleum-free.^83^ As lignin is biodegradable, it takes much more time than the degradation of polysaccharides in soil. According to Thevenot et al., in the laboratory, a soil–lignin mixture takes 13 weeks to 2 years to reduce mass by 16 to 60%, and up to 5 years are required to decrease mass by 48 to 87%.^15^ Many natural polymer-based hydrogels have been prepared successfully by combining lignin with other polymers. Some of these polymers are cellulose, chitosan, alginate, and poly­(vinyl alcohol).^84^ Lignin is second in abundance to cellulose; hence, it serves as a low-cost source for biopolymer-based hydrogels.^85^ Hydrogels based on lignin retain more water than those based on lignin-free hydrogels. According to kinetic studies, over 10 min, lignin-based hydrogels retain 7.2% more water than a hydrogel without lignin.^86^ Extraction of lignin is done through various techniques; thus, various kinds of lignins are produced. Some of these are kraft, lignosulfonate, and organosolv lignin. As various types of lignins are present, a variety of hydrogels based on lignin can be produced. Because of the different extraction techniques employed, their properties differ. As the quantity of β–O–4 bonding in aryl ether is less in kraft lignin, it is not that reactive compared to the other two mentioned. Meanwhile, lignosulfonate lignin dissolves better in water than kraft lignin.^69^ Hydrogels based on lignin can be utilized as a substitute for synthetic hydrogels. Lignin-based hydrogels are environmentally friendly and sustainable hydrogels. Hydrogels from lignin have applications in water purification, petroleum oil drilling, agriculture, soil treatment, biomedicine, drug delivery, and other absorbent materials.^72^

## 8 Synthesis of a Hydrogel Based on Lignin

The procedure for preparing hydrogels based on lignin includes interpenetrating polymer networks, cross-linking, copolymerization, and grafting.^69^

### 8.1 Interpenetrating Polymer Network (IPN)

An IPN is a structure prepared by combining two or more polymers. Among these polymers, at least one is formed or cross-linked within an already formulated network.^69^ This method enables the enhancement of materials with distinctive properties, particularly by embedding lignin within the network. Using semi-interpenetrating or interpenetrating approaches, lignin can be used to form hydrogels that retain their original properties while incorporating new functions.^69,87^ The method for preparing the semi-IPN and IPN is visualized in Figure 8. The key process underlying this approach is free-radical polymerization, in which phenolic hydroxyl groups in lignin generate radicals upon initiation. These generated radicals subsequently react with polymeric chains and monomers to produce a grafted structure, making lignin an intrinsic component of hydrogel.^86^ The formation of an IPN follows numerous stages. At first, a copolymer is formed by the reaction of monomers. Lignin then reacts with these monomers via radical reactions to form a grafted polymer. IPN is fully formed when this lignin-grafted polymer enters a network of monomers, as shown in Figure 8.^88^ Researchers have developed novel techniques for the preparation of these complex hydrogels. A possible method involves forming a thin film, which serves as a foundation for subsequent reactions. Subsequently, polymerization of monomers occurs on the film surface, forming hydrogels with pH sensitivity and strong mechanical properties.^89^

**Figure 8:**
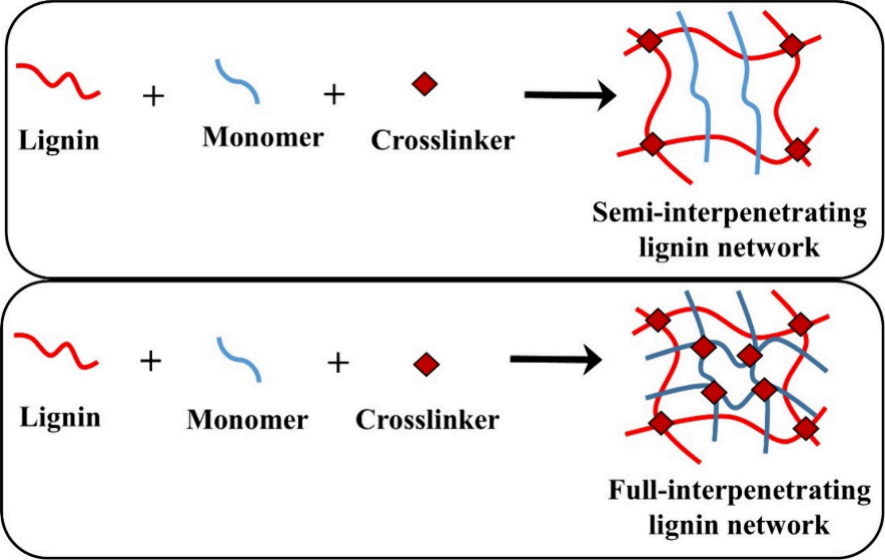
Schematic illustration of lignin-based hydrogel formation via (top) semi-interpenetrating polymer networks and (bottom) fully interpenetrating polymer networks formed through the incorporation of monomers and cross-linker networks.^91^

One example of this technique is the formation of a hydrophilic polyurethane film by solubilizing polyurethane in ethanol, which was then distributed to create a thin layer and allowed to dry. After film preparation, acrylic monomers were added, and ultraviolet light was used to initiate polymerization, yielding a double-network hydrogel with pH sensitivity and hardness. This method has also been applied to prepare lignin-based hydrogels, which combine the natural benefits of lignin with the strength and flexibility of synthetic polymers.^90^ In one investigation, lignin and polyurethane were combined to form a strong lignin-based hydrogel, which was then molded into a framework and treated for 3 days at 60 °C. After it gets dry, the prepared film was dipped in water so it can attain an equilibrium swelling ratio.^89^ The result was a double-network hydrogel that exhibited an enhanced mechanical strength and ease of processing. This synthesized hydrogel could be refined via techniques like casting, fiber spinning, and 3D printing.^88,69^ The technique by which hydrogels acquire the capability to become tough, biocompatible, and multipurpose has become a significant tool for addressing issues related to environmental sustainability and medical innovation.

### 8.2 Cross-linking Copolymerization

The cross-linking present in a polymeric chain can affect its physical characteristics, like elasticity, melting point, viscosity, solubility, toughness, glass transition temperature, and strength.^92^ As the extent to which a polymer chain could rotate is restricted through cross-linking, the cross-linked polymer’s glass transition temperature is higher. Solubilization of the polymer declines as the translational movement of the polymer chain is limited and the molecular weight increases. Although these cross-linked polymers are insoluble, they often absorb a significant amount of solvent, forming a gel.^93^ A hydrogel can be synthesized through physical or chemical cross-linking.

#### 8.2.1 Physically Cross-linked Hydrogels

This type of cross-linking involves physical interactions. These include hydrogen bonding, crystallization, and self-assembly. Hydrogen bonds are formed by molecules having O–H, F–H, or N–H functionalities.

#### 8.2.2 Chemically Cross-linked Hydrogels

Chemical bonding generates the cross-links found in chemically cross-linked hydrogels. When the pendant group reacts chemically, interacts with a cross-linker, or copolymerizes with multifunctional monomers, chemical cross-links are produced.

A lignin-based hydrogel was synthesized in two steps. Lignin was solubilized in aqueous ethanol and then blended with polyethene glycol. The mixture was vaporized to form a film. The produced film was then placed in an oven at 80 °C for approximately 24 h to allow polymerization to occur. This reaction takes place in a solid state and in the absence of a solvent, so it was regarded as sustainable and environment-friendly.^94^

### 8.3 Grafting

To impart the modified material with remarkable properties, polymer refinement is essential. These properties involve thermal stability, multiphase, compatibility, rigidity, flexibility, and physical response.^95^ Refinement of polymer by the grafting method plays a vital part in the formulation development cycle.^96^ The graft polymerization method is exceptionally appealing and compelling. This involves chemically joining one or more covalently bonded polymer side chains to the main chain, which alters the parent polymer’s hydrophilic capacity, rheological characteristics, molecular chain, polymer charges, and aggregation state.^96^ In lignin’s case, reactivity can be modified through grafting it together with unsaturated monomers and several functional groups. Various types of hydrogels can be synthesized for various uses by applying the copolymerization method on grafted lignin with the help of hydrophilic monomers in the presence of a cross-linker. The grafting technique works on the mechanism of radical reaction.^69^ An illustrative example of the grafting method is the synthesis of grafted lignin, prepared by grafting *N*,*N*′-methylenebis­(acrylamide) onto lignin. Then, to prepare a hydrogel in the presence of the initiator (ammonium persulfate (APS)), a variety of useful substances, including organo-montmorillonite and acrylic acid, were copolymerized with the grafted lignin.^69^

Another analysis by Wei et al. reported that a lignin-based hydrogel adsorbent was synthesized via free-radical graft copolymerization. In that process, sodium lignosulfonate, acrylamide, and acryloxyethyltrimethylammonium chloride were used.^97^ For making lignin-based hydrogel, the procedure was as follows: 0.4 g of lignosulfonate was taken in one beaker, and then 1.4 g of acrylamide and 0.036 g of *N*,*N*′-methylenebis­(acrylamide) (MBA) were dissolved in a minimal quantity of deionized water after weighing accurately, and the solution was named “A”. In another beaker, 0.22 g of potassium persulfate (KPS) was solubilized in minimal quantity of deionized water to make solution “B”. Solutions A and B were mixed with vigorous shaking after heating at an invariant temperature in a water bath for 10 to 30 min. Then, in this reaction mixture, 70 μL of tetramethyl ethylenediamine (TEMED) and 0.7 g of acryloxyethyl trimethylammonium chloride (DAC) were added. The reaction mixture was rapidly stirred and polymerized at 40 °C in a thermally stabilized water bath. Thereafter, these continuous reactions produced a lignin-based hydrogel adsorbent (LAD). Finally, the prepared hydrogel was sliced into small segments and soaked in a large volume of distilled water for 3 days. The unreacted substances were removed from the hydrogel by replacing the water twice daily.

## 9 Properties of Hydrogels Based on Lignin

Lignin is a well-known natural-based polymer used to form the biopolymer-based hydrogel shown in Figure 9. Lignin-based hydrogels represent an innovative class of biomaterials with distinct characteristics that make them suitable for various uses, particularly in environmental and agricultural fields.^5^ In the following are the key characteristics of hydrogels based on lignin.

**Figure 9:**
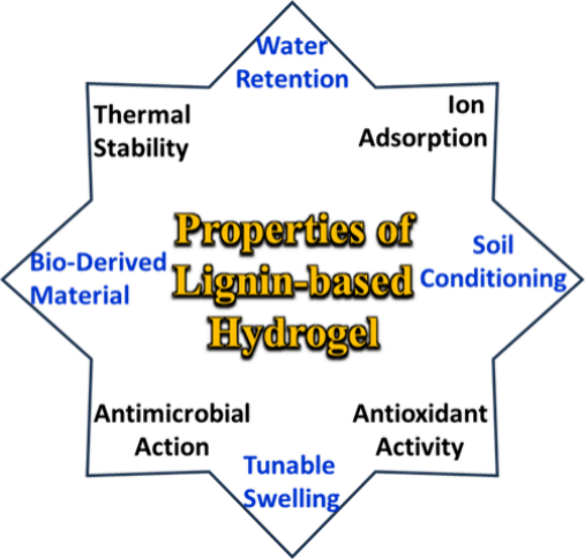
Distinctive features of lignin-based hydrogels, highlighting their versatility and sustainability.^100^

### 9.1 Biodegradability

Hydrogels based on lignin are made from natural polymers, which makes them environmentally friendly and biodegradable. This biodegradable property makes it particularly useful in agriculture. The biodegradation of hydrogels based on lignin is influenced by the density of cross-linking and the numerous phenolic groups present in the hydrogel. Lignolytic fungi, which can break down lignin, attack the phenolic groups of the hydrogel using specific enzymes. Hydrogel can be implemented to protect plants from fungal invasion by reducing the phenol content in it.^98,69^ The cross-link density of a hydrogel can be understood by the occurrence of small pores in it. Hydrogels with high cross-link density are more resistant to microbial attack because fungi and actinomycetes have difficulty accessing these tightly bound structures. Thus, hydrogels with high cross-link density are more effective in resisting microbial degradation as compared to those hydrogels that have lesser cross-link densities.^99^ Because lignin-based hydrogels are environmentally friendly, they are widely used in agriculture to improve oil quality and protect plant roots from microbial damage. The biodegradability and compatibility of these hydrogels can be tested by burying them in soil and measuring their weight loss over time.^99^

### 9.2 Morphology of Surface

Hydrogels based on lignin exhibit a rough surface morphology, in contrast to lignin-free hydrogels such as those composed of pure poly­(acrylic acid), which typically display a smooth, honeycomb-like structure. The implementation of lignin into the hydrogel matrix contributes to an increase in pore size up to a certain threshold. However, exceeding this lignin threshold results in pore closure due to lignin occupying the hydrogel network.^101^ Hydrogels with up to 5% lignin (by weight) tend to have a more porous structure. If the lignin content exceeds 5%, then the porous structure can become distorted, leading to defects and the formation of irregular, larger pores.^102^ When ethanol organosolv lignin is employed as a cross-linker in the synthesis of lignin-based hydrogels, a negative impact on the structure is observed. Lignin-based hydrogels are denser than lignin-free ones, and as more lignin is added, their structure changes from a honeycomb-like pattern to a sheet-like form. Although lignin-based hydrogels have a denser and more uniform network with smaller pores compared to lignin-free hydrogels, they are still expected to have higher pores and a greater surface area.^78^

### 9.3 Water Absorption and Holding

Two main features of lignin-based hydrogels are their capability to absorb and retain water.^103^ These are influenced by two important factors: the hydrogel structure and the functional groups present within it. The first factor is the hydrogel’s structure. The structure includes the pore size and the hydrogel’s surface. Hydrogels with larger pores can absorb more water because they have more available space. Pores that are larger and less densely packed swell more, so they take in more water. The swelling ratio is also affected by functional groups such as carboxyl and hydroxyl. Biomass-based hydrogels, which contain fewer biopolymers, exhibit lower water permeability. Due to this loose network, the hydrogel swells more and holds more water.^104^ Another aspect of swelling and water retention is the functional groups present in the hydrogels. The type of lignin utilized in the formation of a hydrogel based on lignin determines its functional groups. For instance, the use of lignosulfonate can increase the number of active sites, such as free hydroxyl groups. The presence of these groups helps the hydrogel hold and absorb more water. Lignosulfonate can also form a more porous 3D structure, which permits the hydrogel to store more drugs and hydrophobic groups. This enhances the effectiveness of hydrogel for applications like drug delivery.^69,105^ Hydrogels with larger pores and more active functional groups can absorb more water and retain it more effectively. The type of lignin and the hydrogel structure contribute significantly to the ability of the hydrogel.

### 9.4 Mechanical Properties

The storage modulus, dynamic loss modulus, and rheological properties are some of the mechanical attributes of hydrogels based on lignin, which are influenced by the level of lignin available in the hydrogel. Dynamic storage and loss modulus can be respectively represented by *G*′ and *G*″. The stiffness present in the hydrogel is due to *G*′, and energy dissipation present in the hydrogel is due to *G*″.^106^ The amount of lignin present in the hydrogel directly affects the loss and storage moduli. Thus, *G*′ and *G*″ increase with an increase in the amount of lignin.^107^ The hydrogels based on lignin have rigidity in network structure; thus, the value of *G*′ is much more than that of *G*″ in the hydrogel. Due to an increase in *G*′, the lignin backbone gets rigid, which increases the degree of cross-linking, and hence, the network structure also gets rigid. Increased cross-linking in hydrogel and increased *G*″, which increases the content of lignin, both can increase the energy dissipation in hydrogel for the movement of the chain segment. From this observation, it was deduced that on increasing the amount of lignin in lignin-based hydrogel, there can be a significant improvement in mechanical characteristics like storage modulus and loss modulus of hydrogels.^108^ The copolymer solution of thermoresponsive monomer and lignin has lower values of *G*′ and *G*″ at lower temperatures. As *G*′ promotes rigidity to hydrogel, significantly lower values of *G*′ than *G*″ show that the copolymer was in a liquid state. When the temperature was raised to 31–33 °C, the values for *G*′ and *G*″ also increased. However, the value of *G*′ increased far more quickly than *G*″. The hydrogel was finally formed from the copolymer when the temperature reached the crossover point.^109^

Tensile strength is among the mechanical properties held by lignin-based hydrogels. The sol–gel technique was used to prepare both lignin-containing and lignin-free hydrogels. The strength properties of the hydrogel with lignin are excellent. The reason for these properties was the essence of lignin molecules’ ability to interact with each other to generate a “nanoparticle analogue”. This “nanoparticle analogue” allowed lignin to precipitate and form a stiff phase in the lignocellulose hydrogel network. The occurrence of intermolecular H-bonding in hydrogel leads to an association between cellulose and lignin, which would aid in the strength property of hydrogels.^106^

## 10 Why Are Hydrogels Suitable for Agricultural Applications?

Modern agriculture faces numerous serious challenges, including water shortages, soil degradation, and a changing climate. Because of these issues, hydrogels have become increasingly important in the agricultural sector. Hydrogels are materials that can store a specific amount of water and deliver it slowly, which makes them helpful in helping plants grow in harsh conditions (Figure 10).^111^ By developing and improving different types of hydrogels, we can tackle some of the problems farmers face today. Hydrogels can help improve water retention in soil, reduce the need for frequent irrigation, and support plant growth in dry or degraded soils (Figure 11). Below, we explain why using hydrogels in agriculture is becoming more necessary to address these challenges and ensure sustainable farming.^112^

**Figure 10:**
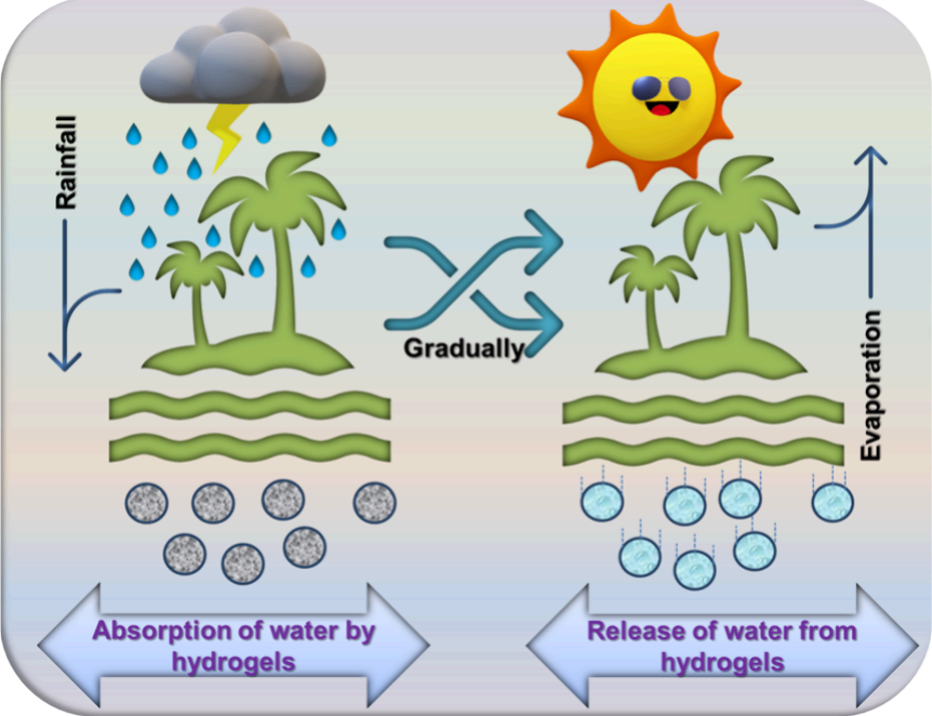
Conceptual schematic illustrating water absorption and release behavior of hydrogels in soil under rainfall and evaporation conditions. The illustration is intended to represent the qualitative mechanism and does not depict quantitative experimental data.^110^

**Figure 11:**
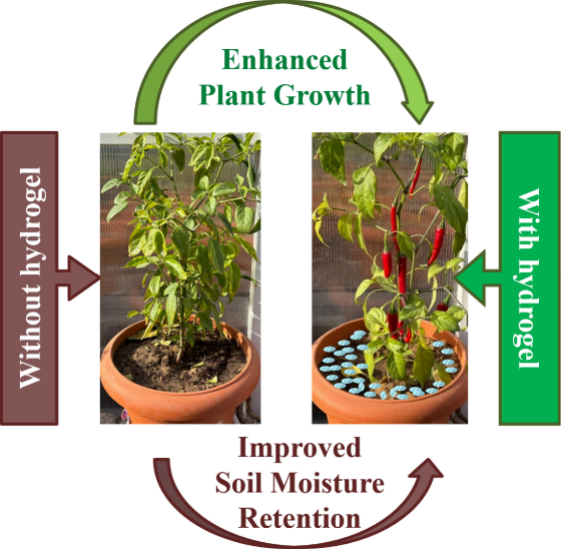
Schematic illustration of how hydrogels enhance plant growth by retaining water in harsh environments.^110^ The figure is intended as a conceptual representation and not as quantitative experimental data.

### 10.1 Water Scarcity

Water scarcity is a significant issue in many regions worldwide. The global agricultural sector is one of the biggest users of freshwater. This places significant pressure on the limited water resources. By applying hydrogels, this concern can be addressed as they can absorb and retain water well beyond their own weight. With the help of hydrogels, water usage in agriculture can be more efficient.^5^ As hydrogels can store large amounts of water and release it slowly, plants can get the required moisture over time without constant watering. This helps conserve water and reduces water loss from common traditional irrigation methods, such as overflow and evaporation. In this way, hydrogels can be helpful to maintain limited water resources by ensuring that less water is wasted and more is used where the growing crops needed.^111^

### 10.2 Soil Degradation

Soil degradation is a major global problem. It affects soil health by causing nutrient loss, soil erosion, and soil compaction. When soil degrades, it becomes less fertile and harder for plants to grow. Soil degradation is a serious threat to agriculture and food production.^111^ Hydrogels can be used to improve soil health and structure in various ways. Hydrogels can enhance the soil’s moisture retention capacity by retaining large amounts of water. This is crucial for plant growth, particularly in arid regions. Hydrogels also help to reduce soil erosion and prevent soil compaction, which can impede root growth and nutrient absorption. By employing hydrogels, the soil can stay healthier for longer. The hydrogel helps maintain nutrient availability for plants, improves water retention, and protects the soil structure. This can slow down the process of soil deterioration and ensure that the land remains fertile and productive for farming.^112^

### 10.3 Drought Resistance

Climate change is leading to more frequent and severe droughts, which negatively affect crop production. Plants are struggling to grow and produce food because of insufficient rainfall or water. In several parts of the world, this has emerged as a major concern. Hydrogels can be helpful for crops to survive during drought conditions by operating as a water reservoir in the soil.^111^ Hydrogels absorb and store water when placed in the zone of a plant’s roots. This stored water is gradually released to the plants as the soil dries, allowing them to access the moisture that they require. This method is effective even during periods of water scarcity.

In periods of low rainfall, farmers can help their crops withstand dry conditions and maintain productivity by using hydrogels. This not only helps plants survive drought but also enhances their resilience and promotes growth under adverse conditions. As a result, hydrogels can play a key role in maintaining crop yields in regions that are affected by water shortages and climate change.^113^

### 10.4 Food Security

Food security has become a significant concern worldwide, as more people require sufficient food to maintain healthy lives. The use of hydrogels in agriculture is one way to ensure food security. Hydrogels can improve crop yields by enhancing crop resilience to adverse conditions and increasing the overall agricultural productivity.

The ability of hydrogels to store water and release it slowly to plants helps them to remain hydrated, especially during dry periods. This also helps the plants absorb more nutrients from the soil. Thus, crops can become more resilient and produce more food. The improvement in the water and nutrient availability for crops by using hydrogels can boost agricultural yields, and this is crucial for feeding the rapidly growing population of the world.^5^

Moreover, hydrogels enable crops to tolerate harsh conditions, such as drought, and enhance their resilience. This indicates that even in such challenging environments, farmers can still produce enough food. The hydrogel plays a vital role in improving food security and supports sustainable farming for the future by increasing both the quantity and the quality of crops.

### 10.5 Sustainable Agriculture

The global population is growing, indicating a need to improve food production. This poses a challenge as we need to identify sustainable ways to produce sufficient crops to feed a growing population without harming the environment. Hydrogels have been proposed as an environmentally friendly solution for managing water and nutrients in agriculture. Hydrogels support sustainable farming by improving the use of water and fertilizers in agriculture. The hydrogel’s ability to absorb large amounts of water and slowly release it to plants helps reduce water loss from drainage and overflow. This indicates that farmers can use less water to maintain crop health (101). Hydrogels also help make fertilizers more efficient by preserving soil nutrients for longer, allowing plants more time to absorb them. This can significantly reduce the demand for excess fertilizers, which often leads to pollution. Hydrogels contribute to a more sustainable approach to agriculture by allowing farmers to use water and nutrients more effectively. Hydrogels help conserve resources and protect the environment. They are ensuring that we can continue to produce enough food for the growing population in a way that is kinder to the environment.^111^

### 10.6 Impact on the Environment

Traditional farming methods sometimes harm the environment. Water pollution, which is caused by the overuse of fertilizers and soil erosion, is a common example of such indirect harm. Traditional practices can lead to long-term damage to ecosystems and reduce soil and water quality. Hydrogels can be helpful in minimizing these negative effects by making farming more environmentally friendly. Hydrogels absorb and retain water and nutrients, releasing them slowly to plants. This implies that less fertilizer would be washed into water resources such as rivers and lakes, thereby helping prevent water contamination. Moreover, hydrogel can improve soil structure and minimize soil erosion, thereby keeping the topsoil intact and rich in nutrients.^114^

Another merit of hydrogels is that many are derived from natural polymers. This indicates that they can decompose on their own over time without leaving harmful residues in the soil. Farmers can also help to protect the environment by applying hydrogels. This can lower the risks associated with traditional farming practices. This signifies hydrogels as a potential tool for reducing the adverse effects of agriculture on the environment.^111^

## 11 Lignin-Based Hydrogels Utilized for Agricultural Applications

One of the most severe environmental stresses that plants may experience is drought, which slows biomass accumulation, growth, and, most importantly, photosynthesis. Water scarcity disrupts the biogeochemical phosphorus cycle and reduces the nutrient availability for plants and crops. These disruptions can significantly impact the productivity of key crops and can result in economic losses. To address these challenges, advances in polymer chemistry have been increasingly applied in agricultural activities. Since the late 19th century, they have attracted significant scientific interest for their potential applications in soil improvement, agriculture, and plant growth enhancement (Figure 12).^5^ Table 2 summarizes previously published research on the use of lignin-based hydrogels in agricultural applications.

**Figure 12:**
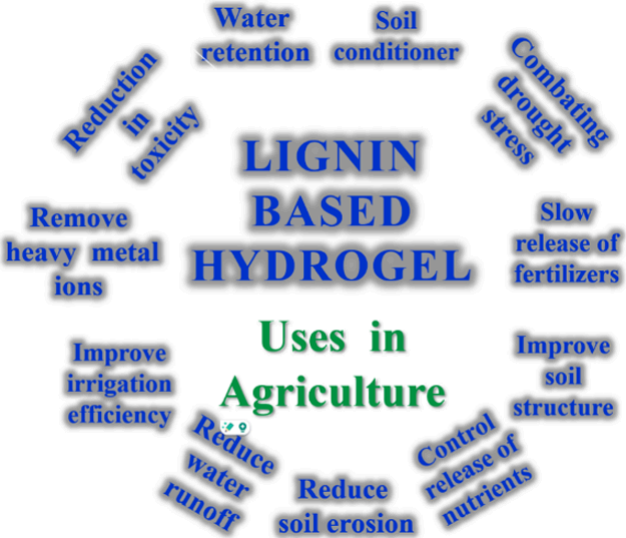
Various agricultural applications of lignin-based hydrogels, highlighting their role in enhancing sustainability and crop resilience under challenging conditions.^115^

**Table 2:** Reported Literature on Lignin-Based Hydrogels Utilized for Agricultural Applications

sr. no.	name of hydrogel	precursor	extraction method	hydrogel synthesis method	application	conclusion	reference
**1.**	lignin/carboxymethyl Sa-son seed gum composite hydrogel	sodium lignosulfonate, potassium persulfate, *N*,*N*- methylenebis(acrylamide), acrylic acid, carboxymethylated Sa-son seed gum	sodium lignosulfonate was acquired from Hefei Tissue Culture Biotechnology	free radical polymerization	1. capable of deactivating Cu(II).	• radical polymerization is done to synthesize a new lignin-based hydrogel.	^118^
					2. lessens the adverse effects from ecosystem.	• good uphold capacity for Cu(II) has been exhibited by the hydrogel in water.	
					3. helps in the treatment of heavy metal pollution.	• prepared hydrogel reduced the content of DTPA (diethylenetriamine pentaacetic acid) extractable Cu in soil.	
						• the amount of Cu decreases in different parts of pakchoi	
**2.**	(Fe_3_O_4_@LH)Fe_3_O_4_-incorporated lignin hydrogel composite	sodium lignosulfonate, acrylamide, maleic anhydride, *N*,*N*,*N*′,*N*′-tetramethylenediamine, *N*,*N*′-methylene bis(acrylamide), potassium peroxydisulfate, nanoferroferric oxide	sodium lignosulfonate was procured from the Shanghai Macklin Biochemical Co, Ltd. (Shanghai, China).	one-pot synthesis method	1. elimination of lead from paddy soil.	• a one-pot synthetic strategy is applied to prepare this hydrogel.	^117^
					2. nutrient levels of the soil are elevated.	• about 16.7–25.4% of total soil Pb is reduced by using hydrogel.	
						• the maximum capacity for adsorption of Pb by hydrogel is 174.2 mg/g.	
						• about 88.3% of the hydrogel is recovered after 90 days of application.	
**3.**	nano-FeS-loaded lignin hydrogel composites	acrylamide, maleic anhydride, sodium lignosulfonate, *N*,*N*′-methylene bis(acrylamide), *N*,*N*,*N*′,*N*′- tetramethylenediamine, potassium peroxydisulfate, ferrous sulfate (FeSO_4_·7 H_2_O)	sodium lignosulfonate was purchased from the Macklin Biochemical Co., Ltd., Shanghai.	cross-linking polymerization	1. bring down the amount of cadmium from the soil.	• excessive recovery rate and good mechanical strength.	^116^
						• remediation of 1 to two times effectively decreases the Cd content from soil and grain.	
						• no bad effect observed on rice biomass or grain yield.	
**4.**	nanoferrous sulfide@lignin hydrogel (FeS@LH)	acrylamide, maleic anhydride, sodium lignosulfonate, *N*,*N*′-methylene bis(acrylamide), *N*,*N*,*N*′,*N*′- tetramethylene diamine, potassium peroxydisulfate, ferrous sulfate (FeSO_4_·7H_2_O)	sodium lignosulfonate was acquired from the Macklin Biochemical Co., Ltd., China, Shanghai	cross-linking polymerization	1. promotes soil fertility.	• the prepared sample shows a high mass recovery rate and good mechanical strength.	^119^
					2. get rid of Cd from paddy soil.	• Cd from soil was reduced by 37.6% and from the plant by 34.5%.	
					3. upgrades the microbial metabolism and restores the function of the microbial community in soil.	• the reduction of Cd from soil was 37.6%, and from plants was 34.5%.	
						• no such h change in soil total potassium and total phosphorus content.	
						• about 96.8% of the mass recovery rate was obtained	
**5.**	lignin-based dual-functional hydrogel	acrylic acid, *N*,*N*-dimethylaminopropylacrylamide, 2,4-dichlorophenoxyacetic acid, 3-indoleacetic acid, 2,2′-azobis(2-methylpropionitrile)	lignin was purchased from Shanghai Aladdin Biochemical Technology Co., Ltd.	free-radical copolymerization	1. controlled release of 3-indoleacetic acid and the herbicide 2,4-dichlorophenoxyacetic acid.	• successful lignin modification confirmed by FTIR spectra and ^31^P NMR.	^21^
						• SEM analysis reveals the porous structure of the prepared hydrogel.	
					2. prevents toxic heavy metals from being absorbed by plant roots.	• major challenges include controlled release of agro-based chemicals and the prevention of heavy metal adsorption.	
**6.**	lignin-based hydrogel	lignin alkali and polyethene glycol diglycidyl ether	lignin alkalis were purchased from Sigma-Aldrich (Mississauga, Ontario, Canada).	free-radical polymerization	1. retains water in the saturated zone of silt loam soils.	• tests were done for superabsorbent property and impact on soil conductivity.	^16^
					2. reduces water stress for plants, lowering the energy needed for water absorption.	• various concentrations of hydrogel were tested on silt loam soil.	
						• hydraulic conductivity for 0.1 and 0.3% (w/w) hydrogel significantly reduced in the saturated zone.	
						• hydraulic conductivity also decreased for 0.3% (w/w) hydrogel in the near-saturated zone.	
**7.**	lignin-based hydrogel	lignin alkali and polyethene glycol diglycidyl ether	lignin alkali for this study was purchased from Sigma-Aldrich (Ontario, Canada)	free-radical polymerization	1. applied as a soil additive to enhance water availability for drought-stressed crops.	• this hydrogel is applied in drought conditions to enhance the growth of maize plants.	^14^
					2. used in drought conditions to boost maize plant height and shoot dry weight.	• leakage of leaf electrolyte and proline can be reduced by the utilization of a hydrogel based on lignin	
						• it leads to the growth of maize plants, which have good water content, phosphorus and biomass.	
**8.**	ferrous sulfide (FeS) nanoparticle lignin hydrogel composite	*N*,*N*-methylene-bis-acrylamide sodium lignosulfonate, acrylamide	sodium lignosulfonate was acquired from Beijing Muhu Additive Co., Ltd., Beijing, China	cross-linking polymerization	1. effectively removes Cd(II) ions from the soil.	• because of the presence of lignin in lignin-based hydrogel, it shows natural environmental stability.	^20^
					2. suitable for long-term soil remediation.	• FeS nanoparticles are added to improve the quality of the soil.	
						• the nanoparticles help increase the hydrogel’s capacity to absorb Cd (II) ions from soil.	
						• nFeS@lh shows high resistance against oxidation.	
**9.**	wheat straw-based hydrogels	chopped wheat straw, carboxymethylated lignocellulosic matrix, citric acid		free radical polymerization	1. effectively elevate the retention of water for sandy soil.	• chopped wheat straw was utilized to prepare lignin-based hydrogel.	^18^
						• the hydrogel showed good mechanical stability, a good enough swelling ratio and reswelling capability after drying.	
						• due to good soil management, hydrogel was used as a novel soil supplement.	
**10.**	lignin-based hydrogel	lignin alkali, poly(ethylene glycol) diglycidyl ether (PEGDGE)	analytical-grade lignin alkali was purchased from Sigma-Aldrich (Mississauga, Ontario, Canada)	free radical polymerization	1. good swelling capacity allows use in saline conditions.	• a number of techniques were used to characterize the formed hydrogel.	^15^
					2. suitable for sodic and drought-affected saline soils.	• insolubility of the product indicated the effective cross-linking between PEGDGE and lignin.	
						• capacity for holding water was especially enhanced for saline, nonsaline and sodic soils, which were impacted by drought.	
**11.**	a biobased hydrogel	lignosulfonate, sodium alginate and konjaku flour	sodium lignosulfonate was purchased from Macklin Biochemical Technology Co., Ltd. (Shanghai, China)	chemical cross-linking	1. enhances photosynthesis in tobacco plants under drought stress.	• after burying for half a year, only 20% of the hydrogel gets degraded.	^120^
						• soil nutrient leaching could be reduced, and water content could be raised by the use of biobased hydrogel.	
						• growth time for tobacco plants could be increased under extreme drought conditions.	
						• it could increase growth time for tobacco plants by 9 to 14 days.	
**12.**	lignin-based polyurethane hydrogels	dimethylol-propionic acid, polyethene glycol, 1,4-dioxane, 2,4-toluene diisocyanate		chemical cross-linking	1. used as a coating substance for slow-release fertilizers.	• a hydrogel was formed by chemical cross-linking between polyurethane ionomers and acetic acid lignin.	^19^
						• the hydrogel had pH-sensitive swelling properties.	
						• the swelling ratio elevated with an increase in pH.	
						• it had agricultural applications, particularly in controlled-release formulations.	
**13.**	lignin-based hydrogels	different technical lignin, poly(ethylene) glycol diglycidyl ether, hydrogen peroxide	Pine Kraft Lignin Indulin at (MeadWestvaco, Charleston, South Carolina, USA)	cross-linking	1. reduces drought stress in various crops.	• two preoxidation methods, in an alkaline medium and in neutral media, were used.	^121^
					2. enhances irrigation efficiency in sandy soils, especially in arid regions.	• various methods like FTIR, ^31^P NMR, Py-GC/MS, and SEM were utilized to confirm hydrogel synthesis.	
						• the humic substance-rich hydrogel had application in soil rehabilitation.	

Hydrogels based on lignin, with their superior water-holding capacity and nutrient retention properties, are particularly well-suited for meeting the water demands of crops that require substantial water input. When incorporated into sandy soils, these hydrogels significantly elevate the soil’s capability to hold water, thereby supporting plant growth. In this context, Zheng et al. prepared lignin-based dual-functional hydrogels through free-radical copolymerization.^21^ These hydrogels are designed to incorporate both conjugated agrochemicals and heavy-metal ligands. The amounts of plant growth regulators, such as 3-indoleacetic acid and the herbicide 2,4-dichlorophenoxyacetic acid, can be adjusted by varying the hydrogel composition. The gradual cleavage of the ester bond controlled the release of these conjugated agrochemicals, which ensured their slow and steady delivery. The release of 2,4-dichlorophenoxyacetic acid from hydrogels effectively regulated lettuce growth, demonstrating the practical effectiveness and feasibility of this system. Furthermore, hydrogels that contain metal-chelating groups such as phenolic OH, COOH, and tertiary amines act as adsorbents for heavy metal ions. This dual functionality of the hydrogel not only enhances soil remediation but also prevents the uptake of hazardous metals through plant roots. The hydrogels demonstrated potential to reduce heavy metal toxicity and improve soil health with significant adsorption capacities for Pb­(II) (60 mg/g) and Cu­(II) (380 mg/g). Two important challenges for agricultural productivity, i.e., the steady release of agrochemicals and the prevention of heavy metal uptake by plants, are effectively addressed. Furthermore, the scientist can explore alternative stimuli-labile bonds, such as hydrazone, imine, and disulfide, for conjugating a broader range of agrochemicals. Moreover, scientists aim to investigate the co-conjugation of various agrochemicals on a single carrier to achieve synergistic effects, which would be a primary focus for future research.

Similarly, the study of Deng et al. aimed to confirm the efficiency of lignin-based hydrogel composites, which are loaded with nano-FeS, for lowering Cd levels in slightly polluted paddy fields and also proposed a practical-scale application strategy.^116^ This is graphically demonstrated in Figure 13. Based on a field experiment conducted across multiple sites, the results showed that applying lignin hydrogel composites loaded with nano-FeS effectively reduced Cd levels in mildly polluted soils and in rice. Whether the lignin hydrogel composites loaded with nano-FeS were used once or twice, they were able to reduce Cd levels to safe limits. Specifically, a single application of lignin hydrogel composites loaded with nano-FeS decreased Cd levels in the soil by 0.42 to 31.72% and in rice grains by 1.52 to 49.11% without affecting rice production. To achieve this, a multisite field study was conducted in southern China. After 94–103 days of application, the lignin hydrogel composites loaded with nano-FeS maintained their structure and elasticity with a recovery rate of 91.90%. This approach offers a novel and effective means of reducing cadmium (Cd) contamination in paddy fields. Applying lignin hydrogel composites loaded with nano-FeS once per rice-growing season is recommended for the best results with even better outcomes when applied multiple times. The lignin hydrogel composites loaded with nano-FeS should be added to the soil over 1–2 weeks following transplanting of rice seedlings and removed before harvesting. The suggested utilization rate is 500 kg per hectare, with one 50 g bag placed per square meter. This demonstrated that nano-FeS-loaded lignin hydrogel composites are an efficient, innovative, and promising technology for remediating Cd-contaminated soils.

**Figure 13:**
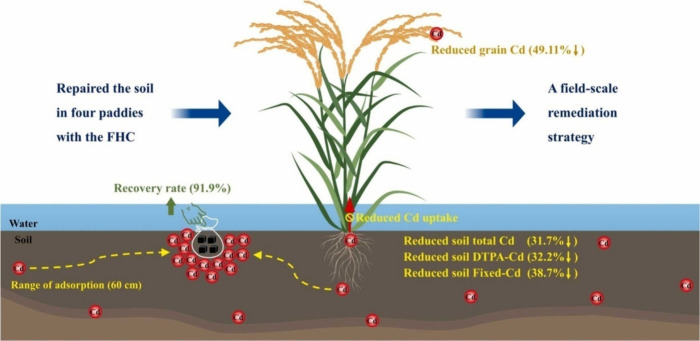
Lignin-based hydrogel composites loaded with nano-FeS in lowering Cd levels in mildly polluted paddy fields and reduced Cd uptake by plants. Reproduced with permission from ref 116. Copyright 2024 Elsevier.

Likewise, Li et al. prepared a nano-Fe_3_O_4_-incorporated stable lignin hydrogel.^117^ Because lead contamination of agricultural soil poses a considerable risk to human health and ecosystems, the prepared hydrogel offers a promising solution. The hydrogel was prepared by combining Fe_3_O_4_ and a hydrogel precursor in a one-pot strategy. This combination enhanced the mechanical strength and stability of the hydrogel’s environment. This study investigated the nano-Fe_3_O_4_-incorporated lignin hydrogel for the elimination of lead (Pb) from paddy soil using soil culture studies and batch adsorption experiments. The adsorption process mainly involves precipitation, complexation, swelling adsorption, ion exchange, and the nanometer effect. Using the prepared hydrogel, soil Pb content declined by 16.7–25.4%, with greater reductions at higher doses and longer application durations. The outcomes for this study were demonstrated by the prediction model’s results from an agricultural field in China, which showed after 112 days of steady use of nano-Fe_3_O_4_-incorporated lignin hydrogel. The content of Pb in moderately Pb-contaminated soil, i.e., 186.55 mg kg^–1^, was dropped below the risk control limit of contamination of agricultural soil, i.e., 140 mg/kg. Simultaneously, the levels of arsenic and cadmium also reduced to 7.1–16.7 and 5.2–10.8%, respectively. As a whole, the use of the nano-Fe_3_O_4_-incorporated lignin hydrogel enhanced soil nutrients and increased total organic and nitrogen matter remarkably. These outcomes highlighted the Fe_3_O_4_ hydrogel as a potential solution for the efficient elimination of lead from contaminated paddy soil. Similarly, the literature reports removal of Cu from soil. Ding et al. examined a major environmental problem: the excessive release of heavy metals, primarily copper. Thus, for the mitigation of copper (Cu) in water and soil, the synthesis of sodium lignosulfonate/carboxymethyl Sa-son seed gum hydrogel was done with the free radical polymerization technique.^118^ The prepared hydrogel based on lignin exhibited effective Cu adsorption (172.41 mg/g) in water and demonstrated a strong capacity for Cu mitigation in soil. Based on the analysis, a mechanism for Cu adsorption was proposed involving electrostatic attraction and surface complexation. These electrostatic attractions occurred between Cu­(II) and −OH/–COOH and ion exchange with −COONa/–SO_3_Na. Moreover, the prepared lignin-based hydrogel (0.7%) effectively depleted 14.1% of the DTPA-Cu in the soil. Additionally, there is a corresponding reduction in the amount of Cu in the roots and leaves of pakchoi of 36.49 and 55.19%, respectively. The presence of a prepared lignin-based hydrogel in soil changed its chemical and physical properties and plays a crucial role in the fixation of Cu in soil Cu. The presence of precipitation and complexation reactions could also decrease the availability of soil Cu. This paper concluded the importance of the prepared sodium lignosulfonate/carboxymethyl Sa-son seed gum hydrogel as a favorable hydrogel for efficiently reducing heavy metals within soil and water. The study suggested the potential use of prepared hydrogel for managing environmental pollution, minimizing negative impacts on ecosystems, and immobilizing Cu in the soil.

Similarly, Wei et al. focus on the potential of nanoferrous sulfide@lignin hydrogel to effectively remove cadmium from both soil and water spinach, and on its impact on soil nutrients and microorganisms.^119^ The study evaluated a prepared hydrogel during a 30-day trial in a paddy field contaminated with cadmium, yielding promising results. After applying nanoferrous sulfide@lignin hydrogel, the concentrations of cadmium in both the soil and the water spinach were significantly reduced. The highest removal of cadmium in soil reached 37.6%, and that in vegetable reached 34.5%. The findings are the effectiveness of prepared hydrogel in minimizing cadmium contamination in both media. Moreover, the nanoferrous sulfide@lignin hydrogel could be reused, with a recovery rate of 96.8%. This indicates the sustainability of nanoferrous sulfide@lignin hydrogel. This hydrogel retained its mechanical strength throughout the process, making it a durable option for environmental remediation. Exceeding the application for the removal of cadmium, the prepared hydrogel contributed to the improvement of soil fertility. In particular, the hydrogel increased important soil health indicators, i.e., total nitrogen content by 16.13% and organic matter content by 13.79%. Although no noteworthy changes were observed in total phosphorus (TP) and potassium (TK) levels, these essential nutrients remained stable, even after treatment. The effective working range of nanoferrous sulfide@lignin hydrogel is its only limit. This hydrogel affected only the 30 cm radius of soil at a rate of 30 g/m^2^. This suggests that it should be applied more extensively or that a larger quantity is required to cover a large area. This hydrogel poses minimal risk to aquatic life. This is shown by the fact that the nanoferrous sulfide@lignin hydrogel did not harm zebrafish larvae. Moreover, the higher microbial activity of the prepared hydrogel in the soil indicated that it removed cadmium as well as promoted the recovery of microbes in the soil. However, the results were promising; further research on the long-term effects of nanoferrous sulfide@lignin hydrogel on soil microbes is needed. Understanding the temporal effects of nanoferrous sulfide@lignin hydrogel on microorganisms is important for ensuring its safe use in the future. Overall, nanoferrous sulfide@lignin hydrogel is verified to be an eco-friendly and productive material for the removal of cadmium-contaminated paddy fields. It also supports improvements in soil fertility and restoration of microbial functions. Thus, a favorable solution is gained for managing heavy metal pollution in agricultural soils.

## 12 Challenges and Future Outlook

Both human actions and natural processes have influenced food security and vegetation production. The food shortage issue has become a worldwide crisis. Crop production has been significantly affected by climate variation. Changes in rainfall patterns, temperature fluctuations, and prolonged drought are among the manifestations of severe climate change. The most vulnerable sector is agriculture, which utilizes the greatest part of freshwater. Thus, it can be predicted that the global population of approximately 6 billion people could face a water shortage by 2050. However, various technologies have been developed to combat drought. These inventions include microsprinklers and drip irrigation. However, the advanced specialized equipment and high costs have constrained their global application. Among several techniques, hydrogels have attracted significant attention as a solution for such conditions. Hydrogels are materials that retain a specific amount of water and can increase the water-holding capacity of soils. This property can enhance crop production and growth. Also, the water runoff and evaporation can be significantly reduced. Among hydrogels, the lignin-based hydrogel has emerged as a promising material due to its properties, including renewability, abundance, and biodegradability. However, lignin-based hydrogel confronts several limitations. These barriers could include limited scalability, high production costs, dependence on biomass, various lignin properties, and the need for advanced modifications to enhance biocompatibility, degradability, and flexibility. However, the mechanical resilience of the lignin-based hydrogel does not provide sufficient swelling and deswelling during repeated cycles. Furthermore, most experiments are constrained to greenhouse or laboratory scales. Thus, there is a critical need to extend the scale from the laboratory to the field. Due to these issues, future research should focus on synthesizing cost-effective, scalable development methods; standardizing lignin sources; implementing green chemistry for further functionalization; strengthening mechanical stability and performance; and conducting long-term field-scale trials. Addressing these challenging issues is important for realizing the full potential of lignin-based hydrogels as a sustainable material to alleviate stress during drought, conserve adequate water, and ensure sustainable global food production.

## 13 Conclusions

Lignin-based hydrogel offers a sustainable approach to mitigate challenges in agriculture, including soil degradation, water scarcity, and food insecurity. As lignin is an abundant and renewable byproduct of lignocellulosic biomass, it facilitates a cost-effective source for the synthesis of hydrogel. Due to the presence of lignin, the lignin-based hydrogel retains a large amount of water, enhances soil properties, and is biodegradable. Notable advancements in lignin extraction methods, structural modification, and hydrogel design enhance the potential of lignin-based hydrogels for crop production and improve resource efficiency. However, field-scale production has been limited by high production costs, variability in lignin sources, insufficient mechanical strength, and the need for advanced chemical modification. Furthermore, most experiments are conducted in controlled greenhouses and laboratory environments; thus, there is a need for further investigation into large-scale validation. For future research, interdisciplinary approaches must be pursued to address scalability, cost-effectiveness of synthesis, standardization for lignin applications, and reliable testing under real agricultural conditions. If these concerns are appropriately addressed, the lignin-based hydrogel could become an excellent material for sustainable agriculture, water conservation, and food security.
